# Measuring Electric Charge and Molecular Coverage on Electrode Surface from Transient Induced Molecular Electronic Signal (TIMES)

**DOI:** 10.1038/s41598-019-52588-6

**Published:** 2019-11-07

**Authors:** Ping-Wei Chen, Chi-Yang Tseng, Fumin Shi, Bo Bi, Yu-Hwa Lo

**Affiliations:** 1 Chemical Engineering program, University of California San Diego, La Jolla, California 92093-0448 United States; 2 Materials Science and Engineering Program, University of California San Diego, La Jolla, California 92093-0418 United States; 3 InnoScouting LLC, Rockville, Maryland 20850-4432 United States; 4 Electrical and Computer Engineering Department, University of California San Diego, La Jolla, California 92093-0407 United States

**Keywords:** Molecular biophysics, Lab-on-a-chip

## Abstract

Charge density and molecular coverage on the surface of electrode play major roles in the science and technology of surface chemistry and biochemical sensing. However, there has been no easy and direct method to characterize these quantities. By extending the method of Transient Induced Molecular Electronic Signal (TIMES) which we have used to measure molecular interactions, we are able to quantify the amount of charge in the double layers at the solution/electrode interface for different buffer strengths, buffer types, and pH values. Most uniquely, such capabilities can be applied to study surface coverage of immobilized molecules. As an example, we have measured the surface coverage for thiol-modified single-strand deoxyribonucleic acid (ssDNA) as anchored probe and 6-Mercapto-1-hexanol (MCH) as blocking agent on the platinum surface. Through these experiments, we demonstrate that TIMES offers a simple and accurate method to quantify surface charge and coverage of molecules on a metal surface, as an enabling tool for studies of surface properties and surface functionalization for biochemical sensing and reactions.

## Introduction

Surface charge density and molecular coverage play important roles in understanding and control of surface chemistries and reactions for many chemical and biomedical applications such as surface coatings, immunoassays, nucleic acid hybridization, etc. Due to chemical potential difference between a solid surface and a solution, the prevailing model suggests that an electrical double layer is formed at the solid-liquid interface^1,2^. It is conceived that the electrical double layer consists of two layers of charge: a Stern layer where the charge is bonded more tightly with the surface atoms in the solid and a diffusion layer where charged ions are highly mobile and their concentrations follow the Boltzmann distribution. If the solid surface is conductive, the total amount of charge in these two layers is counter balanced by the induced charge in the conductive surface to assure charge neutrality of the overall system. All charges in this double layer of the solution phase are called surface charge. Influenced by the sign and density of surface charge, ions or molecules in solution can be attracted to or repelled from the surface via charge-charge or charge-dipole interaction. Hence surface charge density can affect the thermodynamics and kinetics of surface reactions.

Because of the importance of surface charge density to surface chemistry, extensive molecular dynamic simulations have been performed to calculate the surface charge density for solutions of different ionic composition and pH value^3,4^; and many attempts have been made to experimentally measure this and other related quantities^5,6^. So far atomic force microscopy (AFM)^7–11^, surface plasmonic resonance^12,13^, streaming potential^14–18^, and contact angle titration^19–21^ are among the most studied techniques that can produce information related to the surface charge density although none of the existing methods, to our best knowledge, can easily and directly measure the polarity and amount of surface charge in the natural environment where surface reactions take place.

The AFM technique measures surface charge density from interactions between the AFM tip and the surface under the tip; and the technique of surface plasmonic resonance (SPR) measures the effective refractive index due to the distance change between a charged particle and the SPR sensor surface. Both techniques require sophisticated instrument, have low throughput, and rely on the detailed knowledge about the surface properties of the AFM tip or the charged particle^22,23^ which, unfortunately are not always available. On the other hand, the technique of streaming potential measures the voltage difference generated by a pressure driven flow over a charged surface or membrane^14–18^. The measured voltage difference can then be used to obtain zeta potential and the amount of charge in the diffusion layer. However, the amount of charge in the diffusion layer is not equal to the total amount of surface charges according to the double layer model. Hence the streaming potential technique is more suitable for comparing surface properties between different surface modifications and studying zeta potential dependence on pH value and ionic strength of the solution^15,16^. In addition to the above methods, people have also measured contact angles to determine the surface charge density at the liquid/solid interface^19–21^. By combining the Young-Lippmann equation with the Guoy-Chapman model for electrical double layer, the dependence of surface potential and surface charge density on the solution pH value has been studied^19^. However, since the contact angle is highly sensitive to the surface physical and chemical properties, the contact angle titration measurement is quite complex and difficult to obtain reliable results, and often underestimates the surface charge density.

In this paper, we have extended the method of Transient Induced Molecular Electronic Signal (TIMES) reported earlier to directly measure the surface charge for any solution in contact with a conductive surface^24,25^. The results produce the amount and polarity of the surface charge in solution that is in contact with the electrode surface. We integrated TIMES currents over a time period to obtain change in the amount of the surface charge when the test sample is displaced by a reference buffer. We also like to point out that the TIMES signals are generated by ions or molecules that are not permanently adhered to the electrode surface. In other words, if a molecule, charged or not, is permanently anchored to the surface of electrode, it would not contribute to the TIMES signal. Using this property, we can not only measure surface charge density but also surface coverage of molecules that are immobilized on the electrode surface. This is another salient feature for our technique since the knowledge of area coverage of certain molecules such as deoxyribonucleic acid (DNA) capture probes or antibodies is particularly valuable for optimization of the reaction conditions and ensuring reproducible results for any microarrays.

## Results and Discussions

### Device operation principles and test strategy

**Figure 1:**
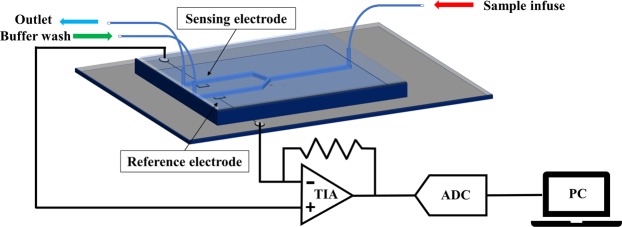
Schematic diagram of device design and TIMES setup.

We have applied the TIMES technique to measure surface charge density for buffers of different concentration (ionic strength), pH value, and buffer types. The TIMES system consists of a microfluidic device with two parallel microfluidic channels that are connected to a single channel via a Y-junction. Within each parallel channel that is 1 mm wide and 30 µm high, there is a platinum electrode connected to the external circuit by a bond wire. The electrode area within the channel is 1 × 1 mm^2^. One of the electrodes is used as the sensing electrode and the other as the reference electrode. Both electrodes are connected to the differential inputs of a transimpedance amplifier (TIA) with a tunable transimpedance (Fig. 1). In the beginning of the experiment, the channel with the reference electrode is filled up with reference buffer, and the channel with the sensing

electrode is filled up with the sample solution. After soaking each electrode in the respective solution for a sufficient amount of time for the system to reach its steady state, we flow the reference buffer into the channel with the sensing electrode at a flow rate of 100 µL/min so that the sample solution in contact with the sensing electrode is displaced by the reference buffer. We call this step the “washing process” and it is during this “washing process” that the TIMES signal is recorded. In other words, we measure the transient current flowing from the sensing electrode into the transimpedance amplifier when the solution above the sensing electrode is switched from the sample solution to the reference buffer.

We can apply the above procedure to measure the absolute amount and polarity of surface charge for essentially any buffer/electrode combinations. The relative difference in the surface charge between the sample solution and the reference buffer can be obtained by integrating the TIMES current signal over the duration of buffer switching from sample solution to reference buffer. We can obtain the absolute amount of surface charge for the sample solution by choosing a reference buffer that has zero surface charge for a certain electrode. Since we know 0.099 M KClO_4_/0.001 M HClO_4_ (pH = 3.4) solution produces zero surface charge with Pt electrode^26^, we can use this buffer and another electrode (e.g. Au) to find the surface charge between the buffer and the new electrode material. Similarly, for a given electrode (e.g. Pt), we can also find the surface charge between a new buffer and the electrode by comparing its signal with the signal from the reference (e.g. 0.099 M KClO_4_/0.001 M HClO_4_) buffer.

In this paper, we firstly used 0.099 M KClO_4_/0.001 M HClO_4_ (pH = 3.4) as the reference buffer to measure the surface charge of 1X PBS (pH = 7.41) in contact with Pt electrode. Then for all other experiments, we used 1X PBS (pH = 7.41) as the reference buffer to measure the surface charge of other solutions of different ionic strength, pH value, buffer types, etc.

The above process can be described in a simple mathematical formula.

1 $$S(t)={\int }_{0}^{t}\,I(\tau )d\tau ={Q}_{sample}-{Q}_{reference}$$

where Q_sample_ is the surface charge in the double layer of the sample solution and Q_reference_ is the corresponding quantity for the reference buffer. Equation (1) also shows that any permanently adhered molecules do not contribute to the signal since only movable charge produces current. Using this important property, we further demonstrated how the TIMES technique can be used to measure surface coverage of molecules anchored to the surface, as described next.

To determine the effect of surface modification by adherent molecules, we have used thiol-modified nucleic acid and 6-mercapto-1-hexanol (MCH) as test molecules. The former forms strong sulfur-platinum linkage, and the MCH molecule also contains a thiol group and is often used as a blocking agent to displace weaker adsorptive contacts between DNA nucleotides and the Pt (Au) substrate to suppress non-specific binding for DNA hybridization experiment. To measure surface coverage by thiol-modified DNA and by MCH, we soaked the sensing electrode in solutions containing different concentrations of thiol-modified DNA. The Pt electrode surface covered by thiol-modified DNA contains fixed charge that cannot be changed by the aforementioned washing process, thus giving rise to no TIMES signal. As a result, if α is the fraction of area covered by the anchored molecule, the magnitude of the TIMES signal in Eq. (1) will be reduced to 1-α times of the signal without molecular coverage. This provides an easy and direct method to measure molecular coverage, a quantity that is critical to the optimization and repeatability for molecular sensing but has not been able to measure till now.

### Measuring surface charge density for 1X PBS buffer using zero-surface-charge buffer as the reference

**Figure 2:**
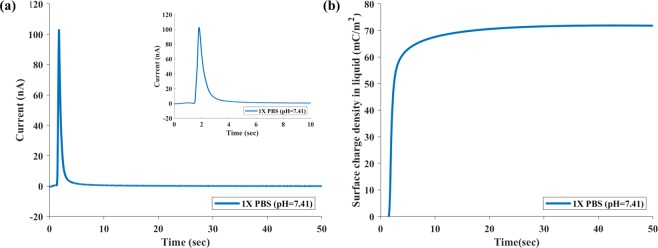
TIMES signal with 1X PBS on the sensing electrode displaced by the zero-surface charge (ZSC) solution. (**a**) TIMES signal. The inset shows the detailed waveform of the current transient. (**b**) Change of surface charge density at the solution/solid interface by integration of the TIMES signal over time. The final value when the system reaches steady state gives rise to the equilibrium surface charge density of the liquid (1X PBS) in contact with a conductive surface (Pt).

Since we used 1X PBS as the reference and washing buffer for most of the experiments discussed in the paper, we first describe the method of measuring the surface charge density for 1X PBS in contact with the Pt electrode. According to Rizo *et al*., the solution of 0.099 M KClO_4_ and 0.001 M HClO_4_ (pH = 3.4) yields zero-surface charge (ZSC)^26^. Therefore, we can obtain the surface charge density for buffer 1X PBS (pH = 7.41) by using ZSC (KClO_4_/HClO_4_) as the reference and washing buffer. Following the procedures described above, we obtained the TIMES signal (Fig. 2a) and the surface charge (Fig. 2b) using Eq. (1) with 1X PBS being the “sample” and ZSC buffer as the “reference”. The result shows that at the 1X PBS/Pt electrode interface, there exists a charge density of 70.67 ± 0.37 mC/m^2^ in the double layers. In the following experiments where we use 1X PBS as the reference and washing buffer, we will add this amount to the results to obtain the actual amount of surface charge density since our method measures the surface charge difference between the sample solution and the washing buffer.

### The effects of ionic strength and pH value on the surface charge density

**Figure 3:**
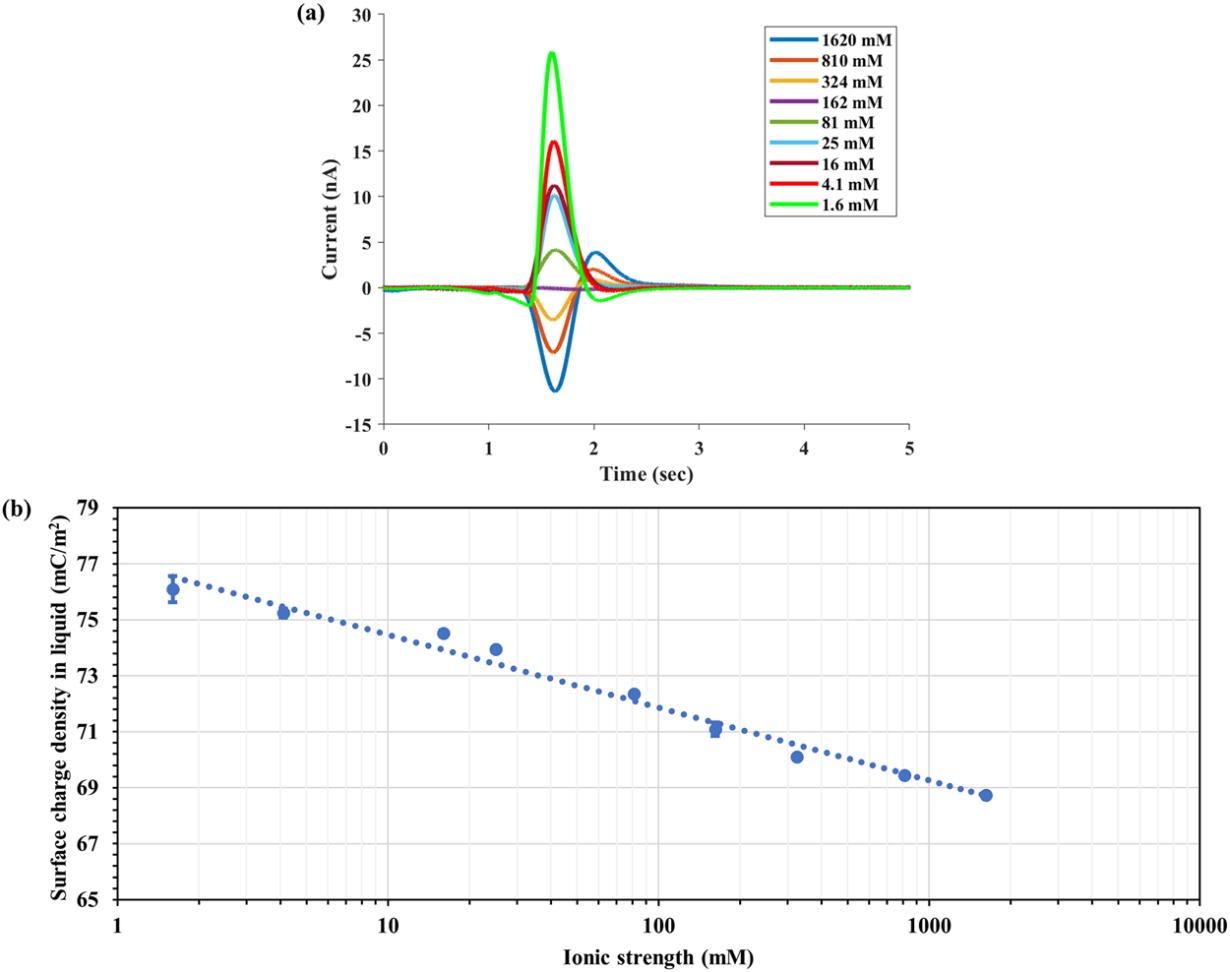
TIMES signals (**a**) and surface charge density (**b**) for different PBS concentration (ionic strength).

The TIMES signals produced by PBS of different concentration (or ionic strength) are shown in Fig. 3a. Applying Eq. (1) with the sample being the PBS of different concentration and the reference (washing buffer) being 1X PBS, we obtain the dependence of surface charge density on the PBS concentration (Fig. 3b). 1X PBS buffer has its ionic strength (IS) of 162 mM and pH value of 7.41. By varying its ionic strength from 1.6 mM to 1620 mM while keeping the pH value the same (by adding a very small amount of HCl or NaOH that did not alter the ionic strength of the buffer), we have found the following relation between the surface charge density and ionic strength:

$$Q-{Q}_{o}=-\,{Q}_{n}\,log(\frac{IS}{I{S}_{o}}),\,{Q}_{n}=2.59\,mC/{m}^{2}$$

It becomes apparent that lower ionic strength produces a greater amount of positive surface charge in the solution in contact with the Pt electrode. However, the effect of ionic strength on the surface charge is rather small since the surface charge density changes from 76.09 ± 0.47 to 68.73 ± 0.06 mC/m^2^ when the ionic strength varies by 1000 times from 1.6 mM to 1620 mM.

**Figure 4:**
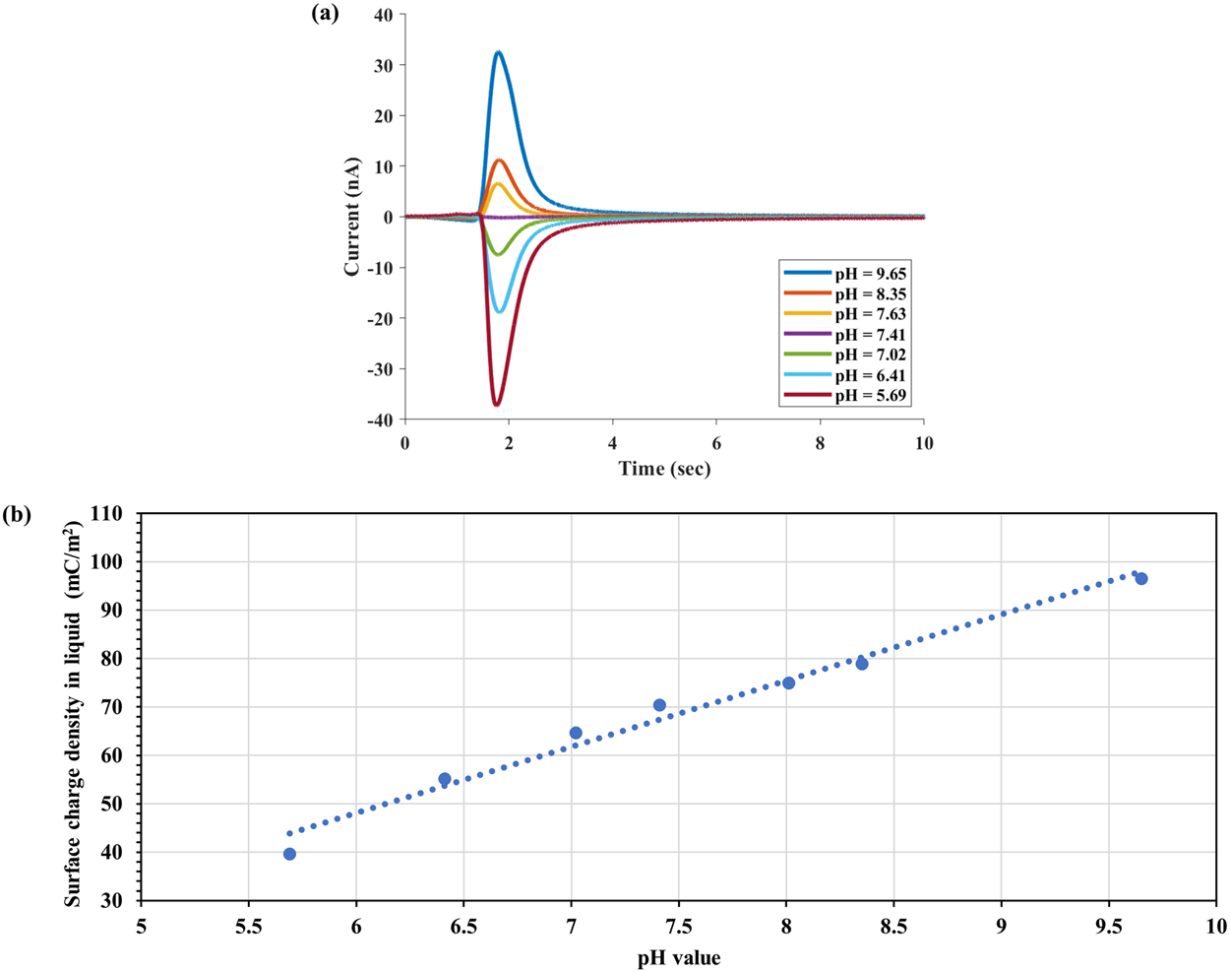
TIMES signals (**a**) and surface charge density (**b**) for different pH value of 1X PBS (IS = 162 mM).

The effect of pH value on the surface charge can be obtained following a similar approach. In this study, we have fixed the ionic strength to 1X PBS (162 mM) and varied its pH value from 5.69 to 9.65 by adding a small amount of HCl or NaOH. Again, using Eq. (1) with 1X PBS (pH = 7.41) being the reference and washing buffer, we have measured the TIMES signals (Fig. 4a) and the surface charge density dependence on the pH value of the buffer (Fig. 4b). From Fig. 4b, we can obtain the relation:

$$Q-{Q}_{o}=-\,{Q}_{m}\,log(\frac{[{H}^{+}]}{{[{H}^{+}]}_{o}}),\,{Q}_{m}=13.67\,mC/{m}^{2}$$

It was found that the surface charge density shows a much stronger dependence on the pH value than the ionic strength. Also, the surface charge density becomes more positive with increasing pH value of the buffer.

### Surface charge density for different buffer types

**Figure 5:**
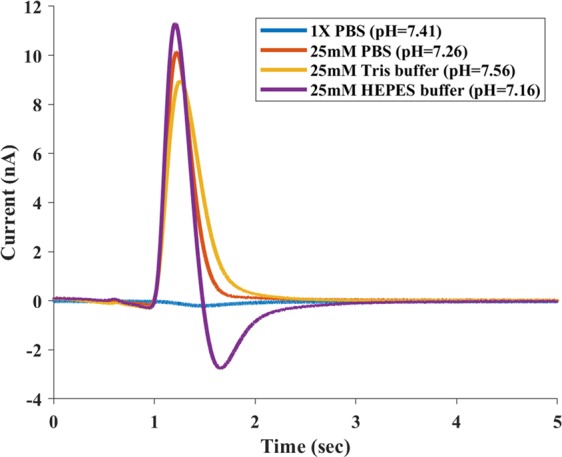
TIMES signals for 25 mM PBS, 25 mM Tris buffer, and 25 mM HEPES buffer with 1X PBS (162 mM) being the reference and washing buffer.

A biological buffer typically consists of a weak acid and its conjugate base to provide a stable pH environment. We have measured the surface charge for some popular buffer solutions for biological samples, including Tris buffer and HEPES buffer. Figure 5 shows the TIMES results when we used 25 mM PBS, 25 mM Tris buffer, and 25 mM HEPES buffer as sample solutions and 1X PBS (162 mM, pH = 7.41) as the reference and washing buffer at room temperature (25 °C). The ionic strength of 25 mM was chosen because it is the preferred concentration for many biological samples. Also noted that for sample solutions under test, we have kept their pH value at their natural state: 7.26 for 25 mM PBS, 7.56 for Tris, and 7.16 for HEPES. After integrating the TIMES signals as before, we have found that the surface charge density for 25 mM PBS, 25 mM Tris buffer, and 25 mM HEPES buffer are nearly the same: 73.94 ± 0.03, 74.19 ± 0.04 and 75.95 ± 0.04 mC/m^2^, respectively.

**Figure 6:**
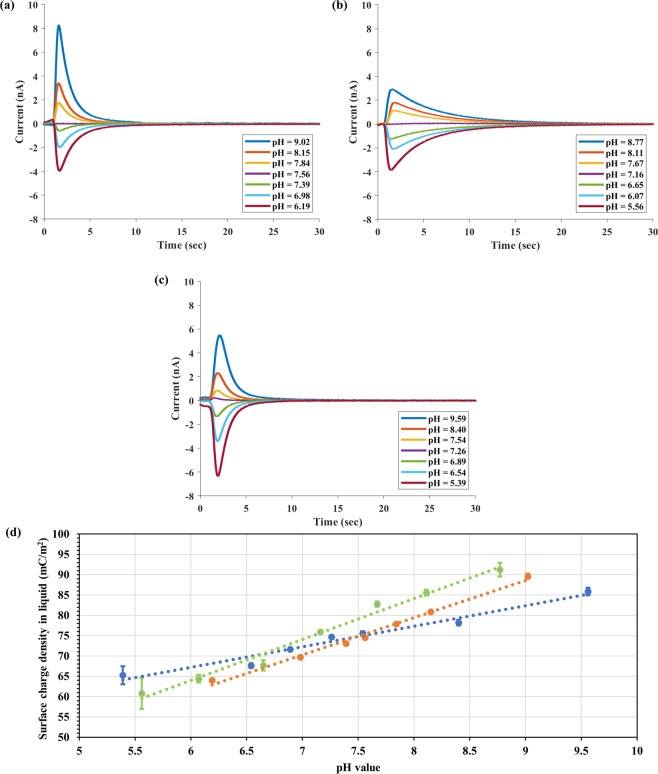
TIMES signals for 25 mM PBS (**a**), 25 mM Tris buffer (**b**), and 25 mM HEPES buffer (**c**) of different pH value. (**d**) pH dependence of surface charge density for 25 mM PBS (blue), 25 mM Tris buffer (orange), and 25 mM HEPES buffer (green) buffers.

Next, we found the pH value dependence of surface charge density for each buffer and the results are summarized in Fig. 6. The TIMES signals in Fig. 6a–c were generated by washing the test samples of different pH value with the same type of 25 mM buffer at its natural pH value (i.e. 7.26 for PBS, 7.56 for Tris buffer, and 7.16 for HEPES buffer). Figure 6d shows the pH dependence of surface charge density for all three buffers.

### Effects of surface modification and surface coverage by immobilized molecules

**Figure 7:**
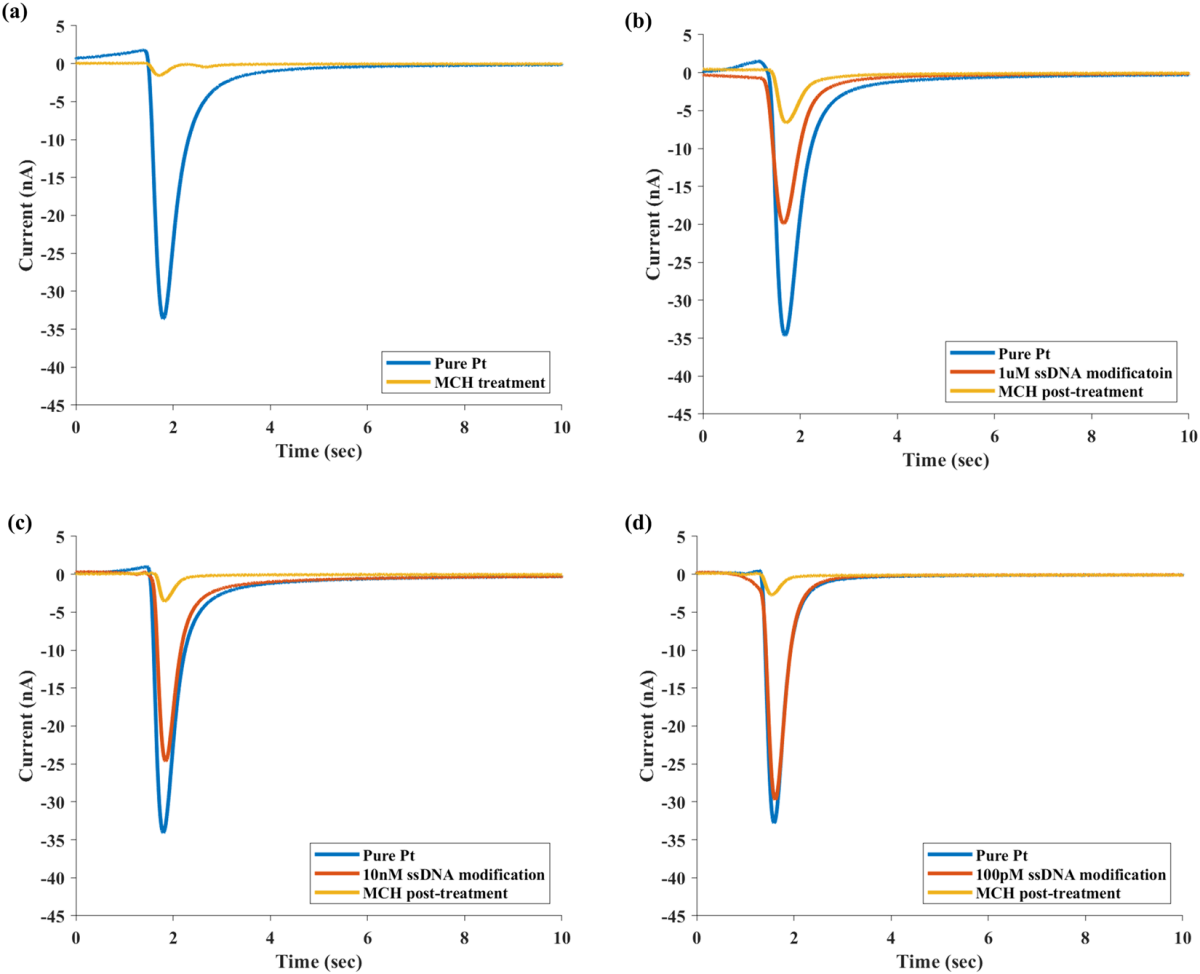
TIMES signals produced by displacing 1X PBS (pH = 5.69) by 1X PBS buffer (pH = 7.41) under different surface modification. (**a**) MCH treatment only, (**b**) 1 µM ssDNA modification followed by MCH treatment, (**c**) 10 nM ssDNA modification followed by MCH treatment (**d**) 100 pM ssDNA modification followed by MCH treatment.

By extending the TIMES method, we can measure the effects of surface modification and the fraction of molecular coverage. Here we have used the concept that any fixed charge created by immobilized molecules on the electrode surface does not contribute to the TIMES signal. Therefore, when a fraction of the electrode surface is covered by immobilized molecules, the magnitude of the TIMES signals decreases. Provided α be the fraction of surface area covered by a type of molecule bonded to the surface, the surface charge density measured by the TIMES signal is expected to be 1-α times of signal without surface coverage. Therefore, by taking the ratio of the integrated TIMES signal with and without molecular coverage, we can obtain the fraction of molecular coverage after surface modification. Such information is highly valuable because quantifying the surface coverage by molecules is essential to assure effective surface treatment and repeatable test results for nucleic acid hybridization, immunoassay, particle capturing, and many surface reactions. In our experiment, we used thiol-modified ssDNA and MCH to demonstrate the ability of measuring surface coverage by adherent molecules. We first tested the surface coverage of MCH as a blocking agent to prevent non-specific binding for sensors of nucleic acid since MCH is supposed to cover any surface area that was not occupied by DNA probes. The sensing electrode in the microfluidic channel was first soaked in 1 mM MCH solution for 3 hours for surface modification. Then the sensing electrode was filled up with 1X PBS with pH = 5.69 as the “sample solution”. When the sample solution was displaced by 1X PBS with pH = 7.41, the TIMES signal was recorded, as shown in Fig. 7a. One can relate surface coverage by MCH to the TIMES signal using the following relations:

2 $${S}_{1}={Q}_{pH5.69}-{Q}_{pH7.41}$$

3 $${S}_{2}=(1-{\alpha }_{MCH})({Q}_{pH5.69}-{Q}_{pH7.41})$$

where S_1_ and S_2_ are the TIMES signals with and without MCH surface treatment and α_MCH_ is the fractional area coverage by MCH molecule. From Eqs (2) and (3), we obtain:

4 $${\alpha }_{MCH}=1-\frac{{S}_{2}}{{S}_{1}}$$

Figure 7a shows the TIMES signals of the above experiment, and the fractional surface coverage for MCH molecule was found to be α_MCH_ = 0.943 ± 0.003, as indicated in the first row of Table 1. The result shows that 94.3 ± 0.3% of electrode surface area has been covered by MCH as an effective agent to prevent non-specific binding in biosensing.

**Table 1:** Fraction of surface coverage by MCH and ssDNA/MCH surface treatments.

Condition	Surface coverage (%)		
	ssDNA	MCH	ssDNA + MCH
1 mM MCH		94.3 ± 0.3	
1 µM ssDNA followed by MCH	48.2 ± 3.3	33.9 ± 3.1	82.2 ± 0.9
10 nM ssDNA followed by MCH	22.6 ± 1.7	69.0 ± 1.6	91.5 ± 0.2
100 pM ssDNA followed by MCH	12.2 ± 0.8	78.8 ± 0.6	91.1 ± 0.7

Next, we performed experiment with bonding of thiol-modified single-strand DNA (ssDNA) probe of different concentrations (1 µM, 10 nM and 100 pM) to the Pt surface. The ssDNA solution was introduced to the sensing electrode and kept overnight to reach the equilibrium state. Then the channel with the ssDNA treated electrode was filled up with the “sample solution” of 1X PBS with pH = 5.69. TIMES signals were recorded when the sample solution was displaced by the reference buffer (1X PBS with pH = 7.41). Following the measurement, 1 mM MCH was introduced to the ssDNA treated electrode as a blocking agent to cover areas uncovered by ssDNA.

Following the same procedure described previously, we measured TIMES signal after MCH treatment. For the ssDNA/MCH treated surface, the signals are expected to follow the relations:

5 $${S}_{4}=(1-{\alpha }_{ssDNA})({Q}_{pH5.69}-{Q}_{pH7.41})$$

6 $${S}_{5}=(1-{\alpha }_{ssDNA}-{\alpha }_{MCH})({Q}_{pH5.69}-{Q}_{pH7.41})$$

where S_4_ and S_5_ are the TIMES signals after ssDNA modification and after MCH treatment, respectively. From Eqs (2), (5) and (6), we can obtain the fractional surface coverage by ssDNA (α_ssDNA_) and by MCH (α_MCH_). The TIMES signals for different ssDNA concentrations and for the MCH treatment that followed the ssDNA surface modification are shown in Fig. 7b–d.

The fractional surface coverage by ssDNA and MCH under different conditions is listed in Table 1. It was found that when the ssDNA volume concentration changes from 1 µM to 100pM, the surface coverage over the Pt surface changes from 48.2 ± 3.3% to 12.2 ± 0.8%. The results approximately follow the logarithmic relation:

$${\alpha }_{DNA} \sim {\alpha }_{DNAo}\,log(\frac{{n}_{DNA}}{{n}_{DNAo}})$$

Another interesting insight is that in all cases, the total fractional surface coverage by ssDNA and MCH is between 82.2 and 91.5% and never reaches 100%. Even with MCH alone, the surface coverage is 94.3% instead of 100%. One possible explanation for this phenomenon is that the surface molecules repel molecules of the same charge polarity. Since thiol-modified DNA contains higher charge density in its sugar backbone than MCH molecule, we have observed higher percentage of coverage by MCH alone (94%) than by ssDNA/MCH combination (82–91%). This hypothesis is also consistent with the observation that the lowest surface coverage (82%) was obtained from the sample having the highest ssDNA coverage^27^. The above findings, made possible by the technique of measuring molecular surface coverage, shed light on the design and optimization of biosensors based on binding with surface probes.

## Conclusions

TIMES method has been proved to be capable of measuring surface charge density with high signal quality. By using the ZSC solution to the Pt electrode as a reference, we were able to measure the actual value of surface charge density for any chosen buffer suitable for biochemical applications. Using the TIMES method and the designed experimental procedures, we have shown quantitatively how the surface charge density is affected by the ionic strength, pH value and type of buffer. Taking advantage of the salient feature that any molecules, charged or not, immobilized on the surface does not contribute to the TIMES signal, we have developed schemes to measure surface coverage for immobilized molecules. We have used thiol-modified ssDNA and MCH molecules as examples to prove the concept. Finally, although in this study we have used time integrated TIMES signals for surface charge and surface coverage measurements, we should mention that rich information is also contained in the temporal waveform of the TIMES signal, which may provide insight about kinetics and charge transport at the solid/liquid interface, as an interesting subject for future study.

## Methods

### Device fabrication

A 1 mm thick glass substrate was cleaned by acetone, methanol and isopropanol (IPA) in sonication and blown-dried by nitrogen gas. On the glass substrate there are two electrodes with 100 nm titanium (Ti) and 200 nm platinum (Pt) formed by sputtering (Denton Discovery 18, Denton Vacuum, LLC, USA) and photoresist (NR9–1500 PY photoresist) (Futurrex, USA) lift-off process. The 100 nm Ti layer was sputtered in 90 seconds under 200 W of power with 35 sccm Argon flow and 2.6 mT chamber pressure. Subsequently, the 200 nm Pt layer was sputtered in 6 minutes under almost the same conditions (200 W, 37 sccm Argon flow, 2.9mT chamber pressure). Each Ti/Pt electrode has an area of 1 mm^2^ within the microfluidic channel and an extended area outside the channel for wire connection to the external instrument. The microfluidic device contained two 1 mm wide, 30 µm high parallel channels connected to single channel by a Y-junction (Fig. 1) and was fabricated by soft lithography process. To create the mold for soft lithography, a layer of 30 µm thick SU8–2050 photoresist (Microchem, USA) was formed by UV lithography to create channel patterns. To transfer the patterns from the mold to polydimethylsiloxane (PDMS, Sylgard 184, Dow Corning, USA), uncured PDMS was poured onto the SU8 mold and cured at 65 °C for 10 hours. After demolding, fluid inlets and outlets were formed at the end of each channel by hole punching. In the final step of device fabrication, the channel-patterned PDMS and the electrode-patterned glass substrate were bonded together after UV ozone treatment. To mount the device onto the system for experiment, the branches connected to the reference electrode and the single channel of the Y-junction were connected to two syringe pumps (PHD Ultra, Harvard Apparatus, USA) which introduced reference buffer and sample solutions into the microfluidic device.

### Surface modification on Pt electrode

In the experiment of measuring surface coverage, the platinum sensing electrode was modified through sulfur-metal bond. The Pt electrode surface was modified by two types of molecules, 6-Mercapto-1-hexanol (MCH) (Sigma Aldrich, USA) and thiol-modified single-strand DNA (ssDNA). The thiol-modified oligos ordered from Integrated DNA Technologies (IDT, USA) were protected by disulfide bond. To reduce the thiol-modified oligos from the disulfide bond, 10 µM oligos solution directly from the stock was mixed with 1 mM dithiothreitol (DTT) in PBS buffer under 4 °C for overnight. Afterwards, the mixture went through a Sephadex column to remove DTT. The deprotected oligos solution was then injected into the sensing electrode channel to immobilize ssDNA on the platinum surface via thiol-Pt bonding. The MCH treatment was performed after the ssDNA modification to occupy the surface area uncovered by ssDNA. The MCH treatment was done by filling the same channel with 1 mM MCH in 25 mM PBS for 3 hours. Between each modification, 1X PBS was introduced into the channel at 100 µL/min for 15 mins to remove any unbonded molecule inside the channel.

### Measurement system set up

The TIMES system detects the current flow between the sensing electrode and the reference electrode in a setup shown in Fig. 1. Both electrodes were connected electrically to the differential inputs of a low-noise transimpedance amplifier (SR570, Stanford Research System, Inc, USA). The output voltage of the transimpedance amplifier was connected to a data acquisition (DAQ) board (USB-6251, National Instrument, USA) that digitized the signal. The output from the DAQ board was then recorded by Labview Signal Express under a sampling rate of 1 kHz.
